# *TBP*, *PPIA*, *YWHAZ* and *EF1A1* Are the Most Stably Expressed Genes during Osteogenic Differentiation

**DOI:** 10.3390/ijms23084257

**Published:** 2022-04-12

**Authors:** Nina Franko, Lucija Ana Vrščaj, Taja Zore, Barbara Ostanek, Janja Marc, Jasna Lojk

**Affiliations:** 1 Department of Clinical Biochemistry, Faculty of Pharmacy, University of Ljubljana, 1000 Ljubljana, Slovenia; nina.franko@ffa.uni-lj.si (N.F.); lucija.ana.vrscaj@ffa.uni-lj.si (L.A.V.); taja.zore@ffa.uni-lj.si (T.Z.); barbara.ostanek@ffa.uni-lj.si (B.O.); janja.marc@ffa.uni-lj.si (J.M.); 2 Clinical Institute of Clinical Chemistry and Biochemistry, University Medical Centre Ljubljana, 1000 Ljubljana, Slovenia

**Keywords:** reference gene, geNorm, NormFinder, BestKeeper, osteogenic differentiation, gene expression, osteosarcoma cell line, RT-qPCR

## Abstract

RT-qPCR is the gold standard and the most commonly used method for measuring gene expression. Selection of appropriate reference gene(s) for normalization is a crucial part of RT-qPCR experimental design, which allows accurate quantification and reliability of the results. Because there is no universal reference gene and even commonly used housekeeping genes’ expression can vary under certain conditions, careful selection of an appropriate internal control must be performed for each cell type or tissue and experimental design. The aim of this study was to identify the most stable reference genes during osteogenic differentiation of the human osteosarcoma cell lines MG-63, HOS, and SaOS-2 using the geNorm, NormFinder, and BestKeeper statistical algorithms. Our results show that *TBP*, *PPIA*, *YWHAZ*, and *EF1A1* are the most stably expressed genes, while *ACTB*, and *18S rRNA* expressions are most variable. These data provide a basis for future RT-qPCR normalizations when studying gene expression during osteogenic differentiation, for example, in studies of osteoporosis and other bone diseases.

## 1. Introduction

Osteoporosis is a common age-related bone disease characterized by low bone mineral density, increased bone fragility, and increased risk of bone fracture upon low-energy trauma. It represents a significant healthcare and economic burden, and can have a detrimental impact on the quality of life and life-expectancy of patients due to bone fractures [1]. With aging populations and new developments in research strategies, osteoporosis has gained more attention in the past decade. Several genome-wide association studies have proposed novel genes and proteins involved in bone biology, followed by in vitro and in vivo functional characterization of candidate genes and proposal of novel mechanisms of regulation of bone cell proliferation, differentiation, and function. This has significantly advanced our understanding of pathological changes in bone mineral density regulation. However, the role of many genes has not yet been explored [2,3].

Most of these studies have been performed on different in vitro cell models of osteoblast origin capable of differentiation and mineralization. This enables close observation of the mechanisms on a cellular level, which is frequently achieved through analysis of gene expression. RT-qPCR is thus the gold standard, a frequently used and widely accessible technique that is applied in almost all studies on osteoblast function and differentiation. However, not all studies account for certain drawbacks of the method or follow MIQE guidelines, which can result in poor inter-assay reproducibility or questionable reliability of results [4].

One of the most important, though frequently overlooked requirements for successful RT-qPCR is choosing the right reference gene against which to normalize the obtained expression data. This normalization accounts for differences in the concentration, purity, and stability of isolated RNA, operator variability, certain pipetting/measurement errors, and potential subjectivity in data analysis. By definition, the internal control reference gene is the gene which expression does not change during the experimental regime under observation (i.e., differentiation). As such, reference genes should be determined separately for each cell/tissue model and treatment. Moreover, according to MIQE guidelines more than one reference gene should be used in order to better account for small changes in expression as well as for operator variability [4].

Unfortunately, most studies involving osteoblast differentiation and mineralization rely on one of the commonly used reference genes, such as *GAPDH* [5,6,7,8,9,10] and *18S rRNA* [11], without proper validation of their suitability for normalization and despite the fact that several studies have shown that these genes are not the most stable during osteogenic differentiation [12,13,14]. Another commonly used reference gene is *ACTB* [15,16,17,18], even though its actin cytoskeleton has been proven to undergo modification during the differentiation process [19], thus raising questions regarding the reliability of the results.

Studies focusing on the identification of reference genes during osteogenic differentiation have thus far been performed on animal cells and human mesenchymal stem cells (MSC). For example, in murine MC3T3-E1 osteoblastic cells, *ACTB*, *HMBS*, and *HPRT1* were the most stable genes, while *GAPDH* and *18S rRNA* were the least stable ones [20]. In rat UMR-106 osteoblasts, *EIF2B1* was the most stably expressed gene, while *ACTB* and *GAPDH* were found not suitable for normalization [21]. For cells of human origin, a structured approach for identification of reference genes has only been performed in primary cells. In induced pluripotent stem cells, *TBP*, *TFRC*, and *RPLP0* were suitable reference genes, while *GAPDH* and *ACTB* were unstable [22]. In bone marrow-derived MSC, *RPLP0* and *GAPDH* or *OAZ1* and *PPIA* were identified as most stable genes depending on the culture type [14]. Another study on bone marrow MSC and umbilical cord blood-derived MSC identified *PPIA*, *HPRT1*, and *YWHAZ* as suitable reference gens [12]. To our knowledge, the only attempt to identify stably expressed genes in human osteogenic cell lines was performed in the MG-63 cell line using the 2^−ΔΔCq^ method, leading to the identification of *RPL13A* as a stable gene [13].

Interestingly, although the osteosarcoma cell lines MG-63, HOS, and SaOS-2 are among the most frequently used osteoblast cell models of human origin, no studies thus far have addressed the stability of reference genes during differentiation of these cell lines. Therefore, the purpose of this study was to determine the most stable reference genes for studies of differentiation and mineralization of MG-63, HOS, and SaOS-2 human osteogenic cell models. This will aid in the selection of more appropriate sets of reference genes in future studies, and provide guidelines on the general stability of internal control genes for use in other osteoblast cell models not addressed in this study.

## 2. Results

The purpose of this study was to evaluate the stability of selected candidate reference genes in order to determine the most stably expressed genes in selected cell lines and possibly find a general set of genes suitable for other cells of osteogenic origin during differentiation. To obtain this, MG-63, HOS, and SaOS-2 cell lines were differentiated for up to 35 days and mRNA samples were collected every 7 days of differentiation. Gene expression of ten selected candidate reference genes was measured using RT-qPCR and corresponding quantification cycle (Cq) values were analysed by three types of commonly used software, geNorm [23], NormFinder [24], and BestKeeper [25], which calculate the most stably expressed gene(s) in each set based on specific statistical algorithms. The results were used to obtain comprehensive rankings of the most stable genes for each cell line and a suggested gene ranking for cells of bone origin in general.

### 2.1. Osteoblast Differentiation

**Figure 1:**
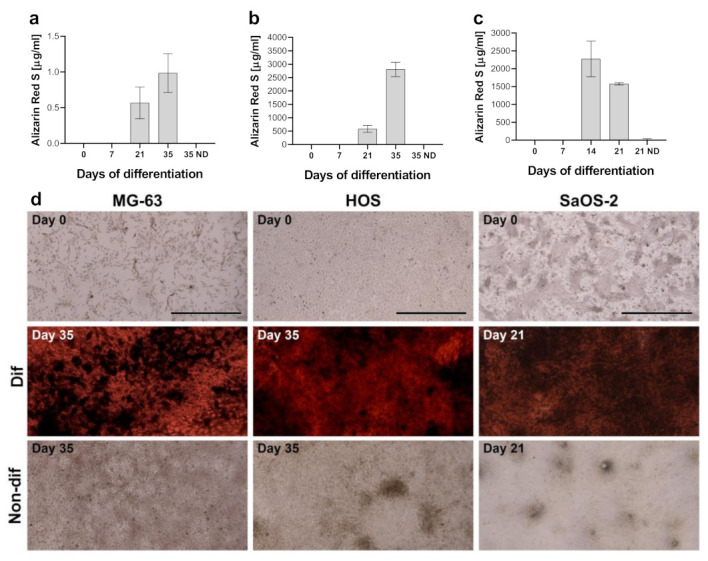
Exposure to differentiation media-induced mineralization in (**a**) MG-63, (**b**) HOS, and (**c**) SaOS-2 cells as determined by Alizarin Red S staining. Average deviation and standard deviation of spectrophotometric measurements of Alizarin Red S staining are shown for three independent experiments. (**d**) Representative microscopic images of cells at the beginning (day 0) and end (day 21 or 35) of differentiation procedure for differentiated and non-differentiated cells. Scale bars correspond to 1 mm.

To induce osteogenic differentiation and mineralization, MG-63, HOS, and SaOS-2 cells were exposed to osteogenic differentiation media for up to 35 days. SaOS-2 cells were only differentiated for up to 21 days, as the cells began to detach with longer culture times. To confirm mineralization, cells were stained with Alizarin Red S and observed under a microscope. As expected, the amount of stained and mineralized extracellular matrix increased in a time-dependent manner (Figure 1d and Figure S1), which was further confirmed by spectrophotometric analysis of the stained samples (Figure 1a–c). The lowest degree of mineralization was observed in MG-63 cell line, while HOS and SaOS-2 showed considerable Alizarin Red S staining. In the non-differentiated controls, no mineralization of extracellular matrix occurred even after 35 days of culturing. This confirmed that a differentiated functional osteoblast cell type was obtained, allowing us to analyse gene stability during the differentiation process.

### 2.2. Candidate Reference Gene Selection and Primer Validation

To determine the optimal internal control genes for study of differentiation processes, ten candidate reference genes were selected (see Table 1). The selected genes mostly belong to different functional classes, and were selected based on most stably expressed genes detected in other similar studies. The performance of all primers was validated for each cell line. Amplification efficiencies for each gene were calculated from standard curves and ranged from 92.6% to 106.7%, with corresponding correlation coefficients (R^2^) from 0.9956 to 1 (Table 2). All primers had efficiencies within the recommended range (100 ± 10%) [26]. For each primer set only one melting curve was detected, confirming primer specificity (Figure S2). These findings confirmed that the designed primers were suitable for use on all three selected osteoblast cell lines.

### 2.3. Analysis of Stability of Candidate Reference Genes

**Figure 2:**
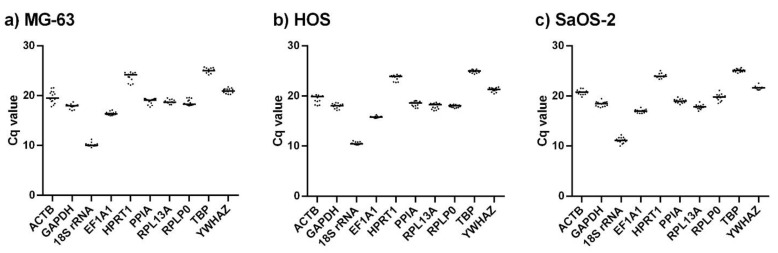
Data distribution and average of measured Cq values at different time points for each tested gene in (**a**) MG-63, (**b**), HOS and (**c**) SaOS-2 cell lines obtained with RT-qPCR.

To determine the expression stability of the selected genes, their expression was determined at different time points during osteogenic differentiation in all three cell lines. Expression profiles were first evaluated by descriptive statistics. In all cell lines, each gene reached the detection threshold at a comparable Cqs, indicating comparable levels of expression in each cell line (Figure 2). The lowest Cq values were from 10.16 ± 0.39 to 11.16 ± 0.59 for *18S rRNA*, while the highest were from 24.94 ± 0.30 to 25.08 ± 0.31 for *TBP*. Figure 2 shows the distribution of Cq values for each gene at different differentiation time-points and provides an overview of the stability of the measured values during differentiation. Further analysis of stability was performed using three commonly applied statistical algorithms, geNorm, NormFinder, and BestKeeper.

#### 2.3.1. GeNorm Analysis

**Figure 3:**
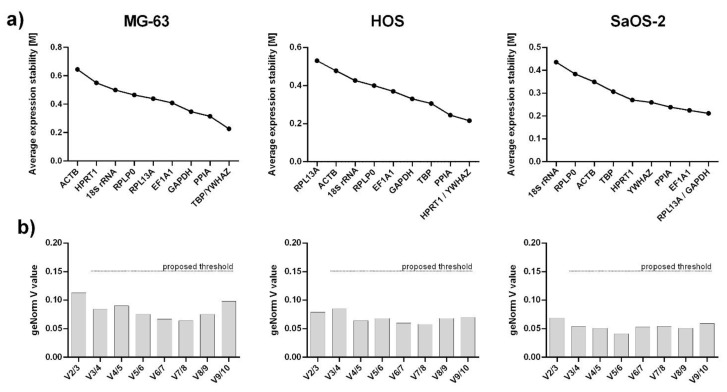
Stability of candidate internal control genes as determined with geNorm. (**a**) Gene ranking based on M value as a measure of gene stability (average pairwise variation of a particular gene with all other control genes; lower M value indicates higher stability). (**b**) geNorm’s pair-wise variation analysis for determination of minimal number of required genes for reliable normalization.

geNorm’s pair-wise approach ranks genes based on their M value, where a lower M value represents higher gene stability. As seen in Table 3, all calculated M values were < 1.5, therefore, all genes in every cell line are considered stable. However, the lowest M values, and therefore the most stable genes, were *TBP* and *YWHAZ* for MG-63 (M = 0.227), *HPRT1* and *YWHAZ* for HOS (M = 0.216), and *RPL13A* and *GAPDH* for SaOS-2 (M = 0.212). On the other hand, the highest M values (the least stable genes) were attributed to *ACTB* in MG-63 (M = 0.645), *RPL13A* in HOS (M = 0.531), and *18S rRNA* in SaOS-2 (M = 0.436) (Table 3 and Figure 3a).

The pairwise variations analysis showed that the pairwise value V2/3 in every cell line is already below 0.15 (0.113 for MG-63, 0.079 for HOS, 0.069 for SaOS-2); therefore, the normalization to two most stable genes is sufficient and the addition of the third normalization gene does not contribute significantly to the reliability of the results (Figure 3b).

#### 2.3.2. NormFinder Analysis

NormFinder uses a model-based approach by which it ranks genes according to their stability value. Again, the lowest stability value is attributed to the most stable gene. This algorithm identified *PPIA* as the most stable gene in both MG-63 and SaOS-2 (0.079 and 0.054, respectively). These two cell lines also share the least stable gene *18S rRNA* (0.248 for MG-63 and 0.237 for SaOS-2). In HOS cell line, NormFinder identified *YWHAZ* as the most stable (0.114) and *RPL13A* as the least stable gene (0.478) (Table 3).

#### 2.3.3. BestKeeper Analysis

BestKeeper ranks genes’ stability based on their standard deviation (SD_Cq value_; ±Cq), coefficient of variation (CV_Cq value_; % Cq), and correlation coefficient (r). MG-63 and HOS share *EF1A1* as a gene with the lowest SD (0.259 for MG-63 and 0.124 for HOS) and *ACTB* as the gene with the highest SD (1.011 for MG-63 and 0.666 for HOS). In SaOS-2, *YWHAZ* exhibits the lowest SD (0.209) and *RPLP0* the highest SD (0.514). As BestKeeper’s requirement for stable gene is SD < 1, all genes, with the exception of *ACTB* in MG-63, were considered stable.

Ranking based on CV reveals that the top/bottom ranked genes are identical or at least closely related to their position based on SD ranking. The lowest CV exhibited *TBP* (1.387), *EF1A1* (0.786), and *YWHAZ* (0.964) for MG-63, HOS, and SaOS-2, respectively. The highest CV was observed in *ACTB* for MG-63 (5.125) and HOS (3.426) and in *18S rRNA* in SaOS-2 (4.080) (Table 3).

Based on the correlation coefficient, the most stably expressed genes are *PPIA* in MG-63 (0.933) and SaOS-2 (0.920) and *HPRT1* in HOS (0.907). *18S rRNA* exhibits the lowest correlation in MG-63 (0.063) and HOS (−0.121), while *TBP* (0.272) is ranked as the least stable in SaOS-2.

#### 2.3.4. Comprehensive Ranking

As the three applied algorithms use different logic to determine gene expression stability, each of them provided a different ranking based on the same Cq values for each cell line (Table 3). As none of these approaches is considered more reliable than the others, we included all of them and performed a comprehensive ranking using two approaches: the geometric mean of the ranks and NormFinder’s algorithm for estimating variability between subgroups. Based on the geometric mean comprehensive ranking, the most stably expressed gene in MG-63 is *TBP*, while *YWHAZ* is the most stable in both HOS and SaOS-2 (Table 3). The least stably expressed are *ACTB* in MG-63, *RPL13A* in HOS, and *18S rRNA* in SaOS-2. On the other hand, NormFinder’s model-based algorithm identified *GAPDH* and *EF1A1* as the best combination for normalization (Table S1).

As certain genes were consistently ranked as more stable in all three observed cell lines, we wanted to determine whether certain genes would show general stability across different cell lines, making them suitable for use as a starting point in experiments involving osteoblast differentiation. We therefore combined the comprehensive ranking obtained for each cell line in this study and calculated the geometric means of the ranks for each gene. Using this approach, *YWHAZ* and *TBP* were identified as most stable, closely followed by *PPIA* and *EF1A1* (Table 4).

To test our assumption and confirm the ranking obtained in this study, we collected the data from other similar studies where gene stability in cells of bone or osteoblast origin was evaluated [12,13,14,20,21,22,27]. Stability ranks obtained in these studies were combined with our data and a comprehensive ranking of each gene was determined using the geometric mean of the ranks. As in our study, *PPIA, TBP*, and *YWHAZ* ranked as the most stable and *ACTB* as the least stable gene (Table S2).

### 2.4. Normalization of Genes Involved in Osteogenic Differentiation against Selected Reference Genes vs. ACTB

**Figure 4:**
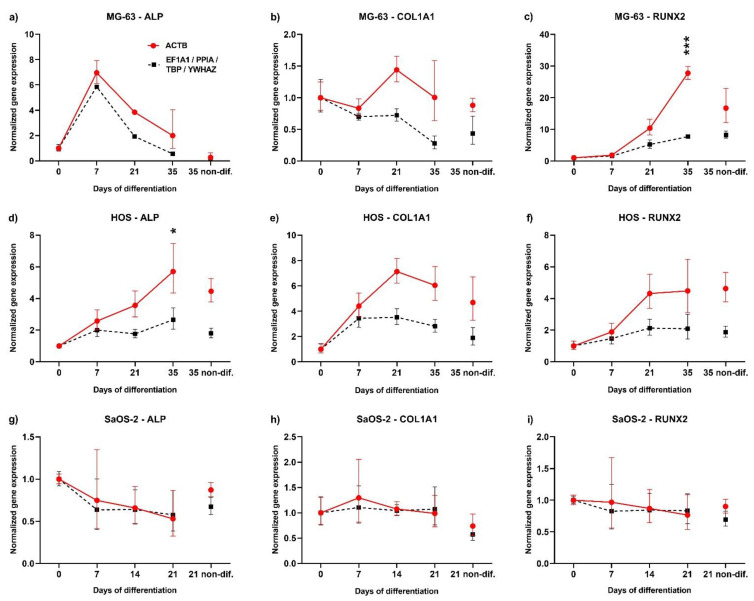
The choice of reference gene can impact obtained gene expression. MG-63 (**a**–**c**), HOS (**d**–**f**), and SaOS-2 (**g**–**i**) cell lines were differentiated and expression of *ALP* (Alkaline Phosphatase) (**a**,**d**,**g**), *COL1A1* (Collagen type I alpha 1 chain) (**b**,**e**,**h**), and *RUNX2* (Runt-related transcription factor 2) (**c**,**f**,**i**) differentiation markers was analysed at given time points using RT-qPCR. The relative quantity of each marker gene was normalized to *ACTB* (red) or to geometric mean of *TBP*, *PPIA*, *YWHAZ*, and *EF1A1* (black) internal control genes. Results represent the mean and standard error of relative expression for three independent experiments. Statistical differences between the two normalizations were analysed using two-way ANOVA and are denoted as * *p* ≤ 0.05 and *** *p* ≤ 0.001.

To determine the influence of the reference gene, we determined the expression of three commonly used osteogenic differentiation markers, Alkaline phosphatase (*ALP)*, Collagen Type I Alpha 1 (*COL1A1)*, and Runt-related transcription factor 2 (*RUNX2)*, in each cell line: the expression data was normalized using the geometric mean of the relative quantities of *TBP*, *PPIA*, *YWHAZ*, and *EF1A1* genes or *ACTB* gene (Figure 4). Although the shapes of the obtained graph curves are similar and show the same trends, normalization against ACTB significantly increased the normalized expression of all three markers in MG-63 and HOS cells. Interestingly, there were no significant differences in expression obtained in SaOS-2 cells, and the expression of differentiation markers remained mostly stable during the differentiation process, suggesting that these cells were already finally differentiated and only underwent mineralization. This indicates that the choice of reference gene can strongly influence the fold increase of the observed expression changes after normalization.

## 3. Discussion

Differentiation of osteoblast-like cells is a highly complex multistep process of cell maturation during which the cells continuously deposit mineralized extracellular matrix [28]. In vitro differentiation is stimulated with dexamethasone to enhance expression of the transcription factors, L-ascorbic acid (Vitamin C) to increase collagen type I secretion, and β-glycerophosphate as a source of phosphate for hydroxyapatite formation and additional stimulation of signalling pathways [29]. The differentiation process can be followed by detecting the mineralization of the extracellular matrix using Alizarin Red S staining, as well as by following the expression of differentiation markers such as osteocalcin, ALP, RUNX2, COL1A1, etc. [22,28,30]. When measuring differentiation markers using RT-qPCR, their expression levels should be carefully normalized to reference genes that remain stably expressed during the differentiation process.

We exposed three commonly used human cell lines of osteogenic origin, MG-63, HOS, and SaOS-2, to osteogenic differentiation media in vitro. MG-63 and HOS cell lines were treated for 35 days, while SaOS-2 differentiation was terminated at day 21 due to the spontaneous detachment of cells from the plates. Time-dependent increase in mineralization was observed in each cell line (Figure 1 and Figure S1). Despite being cultured for only 21 days, SaOS-2 exhibited extensive mineralization even at this time point. While HOS and SaOS-2 cell lines were extensively mineralized as judged by Alizarin Red S, MG-63 cells showed only modest mineralization at day 35 when examined spectrophotometrically. However, when observed under the microscope deposition of minerals was clearly seen and significantly different from the non-differentiated control. Therefore, by the last day of culture all of treated cells exhibited a differentiated/mineralized phenotype.

To determine the best reference genes for studying osteoblast differentiation, we first chose ten genes that are either commonly used as reference genes (*GAPDH*, *18S rRNA*, *ACTB*) or have been found to be the most stable in other similar studies addressing gene stability in cells of bone/osteoblast origin (*PPIA*, *EF1A1*, *RPL13A*, *RPLP0*, *TBP*, *HPRT1*, *YWHAZ*). Expression profiling of the selected genes revealed that all of the genes were comparably expressed in all cell lines. While a majority of the genes exhibited similar Cqs (approximately 15–20), *18S rRNA* had significantly lower Cq (~10) whereas *HPRT1* and *TBP* Cqs exceeded 20. These differences arise from differences in basal expression of the genes, and could be avoided by modifying the amount of starting material. However, we chose to keep the amount of starting material constant in all experiments in order to avoid differences in sample preparation possibly leading to higher inter-experimental variability.

We applied geNorm, NormFinder, and BestKeeper software the selection of the appropriate reference gene. Each of these software types uses a different statistical algorithm; therefore, their integrated use might represent a way to avoid discrepancies in rankings. Such approach is a commonly used for determining the most stable genes [31,32,33]. As expected, we observed differences in the top-ranked genes between the different algorithms. However, in general, certain genes consistently ranked as more stable while others were consistently among the least stable, with the exceptions attributed mainly to BestKeeper’s algorithm and ranking. For example, *TBP* in SaOS-2 drops from second place in BestKeeper’s SD and CV ranking to the tenth place in correlation coefficient (r) ranking. This parameter describes the correlation between a chosen candidate gene and the geometric mean of all housekeeping genes (BestKeeper index). It has been previously reported that correlation coefficient describes the stability of the gene better than SD, as the correlation coefficient correlates each gene with the BestKeeper index and SD can vary with the amount of input material in each sample [26,34]. Not surprisingly, the lowest correlation index was attributed to *TBP*, which exhibited the highest Cq values (~25), and *18S rRNA*, which had the lowest Cq values (~10). On the other hand, the lowest-ranked genes by BestKeeper’s correlation coefficient agree with the rankings by geNorm and NormFinder. Generally, the least stable genes correlate well between the algorithms.

The geNorm algorithm ranks genes based on their M value, which is inversely proportional to their stability. However, this algorithm cannot distinguish between the two most stable genes, and it is biased towards genes which could be co-regulated. Taking this issue into account, NormFinder and BestKeeper are considered to be more robust, as their algorithms are less subject to co-regulation [24,25]. When comparing the top-ranked genes by NormFinder and geNorm, similar rankings can be observed. At this point, it must be noted that based on the cut-off values of the chosen algorithms, namely, geNorm’s M value < 1.5 and BestKeeper’s SD < 1, all genes were considered stable with the exception of *ACTB*’s SD in MG-63 (SD = 1.011). The differences in ranking values between consecutively-ranked genes are relatively small, showing similar degrees of stability. This outcome is not surprising, as the initial selection of most of the candidate genes was based on the findings of previous studies which identified them as stable in differentiation processes of cells of similar origin [13,20,21,22,34,35,36,37].

Of the three algorithms, geNorm is the only one that proposes the number of genes required for normalization. Using pairwise variation analysis, it calculates the V value which describes the relevance of the additional gene to the normalization process. It is proposed that 0.15 is a cut-off value below which an additional gene does not contribute significantly to normalization [23]. In every cell line, geNorm calculated that two genes were sufficient for normalization. Using a comprehensive ranking by geometric mean which integrated data from the different algorithms, we chose two appropriate reference genes for normalization during osteoblastic differentiation in each cell line; these were *TBP* and *PPIA* in MG-63, *YWHAZ* and *EF1A1* in HOS, and *YWHAZ* and *PPIA* in SaOS-2.

Even though MG-63, HOS, and SaOS-2 cells were of human osteosarcoma origin and were exposed to identical differentiation stimuli, different genes were identified as the most stable in these cell lines. These differences likely arise from their internal characteristics, e.g., differentiation stage and genetic instabilities, which offer different starting point for differentiation and mineralization. As observed in the comparison of the rankings of different algorithms for each cell line, certain genes were consistently ranked as more stable when comparing different cell lines. As we wanted to propose a group of stable reference genes for reliable use in studying osteoblast differentiation and mineralization, we compared NormFinder’s inbuilt algorithm for comparison of different groups to the geometric mean of all compared ranks for each gene in this study.

NormFinder’s model-based algorithm identified *GAPDH* and *EF1A1* as the best combination for normalization (Table S1). However, *GAPDH* exhibits relatively high SDs and CVs (especially in HOS and SaOS-2 cell lines) and *EF1A1* shows unfavourable correlation with the BestKeeper index. Additionally, selection of the best genes for normalization by NormFinder has previously been shown to be unreliable [38]. On the other hand, the geometric mean of the ranks for all three cell lines identified *YWHAZ* and *TBP* to be the most stable genes (Table 4).

Additionally, we combined our data with comprehensive rankings from other studies, selecting only cells of bone or osteoblast origin (Table S2) [12,13,14,20,21,22,27]. This ranking is comparable with the overall rankings obtained in this study, suggesting that *YWHAZ*, *TBP*, *PPIA*, and *EF1A1* are stably expressed during osteoblast differentiation. Small differences in their geometrical means confirm that their expression stabilities are very similar.

To assess the selection of the most stable internal control genes, the same samples were analysed for expression of the differentiation markers *ALP*, *RUNX2*, and *COL1A1* (Figure 4). The obtained expression data (relative quantity) were normalized to the *ACTB* reference gene (a commonly used internal control) and to the geometric mean of the four internal control genes detected as most stable in this study (*TBP*, *PPIA*, *YWHAZ*, *EF1A1*). The results showed that for the MG-63 and HOS cell lines normalization to *ACTB* can considerably increase the obtained values, especially in later differentiation stages. This suggests that *ACTB* expression decreases during differentiation compared to the geometric means of expression of *TBP*, *PPIA*, *YWHAZ*, and *EF1A1*. Interestingly, in SaOS-2 cells both normalizations showed similar results. Furthermore, there were no changes in expression of observed differentiation markers despite significant mineralization, a phenomenon which has been observed previously [39,40]. This indicates that the SaOS-2 cell line consists of terminally differentiated osteoblast cells and that differentiation media only induces mineralization (Figure 1 and Figure S1). The lack of changes in the differentiation state of these cells could explain the stability of *ACTB* expression during mineralization as well. These results indicate that the choice of internal control can have a significant impact on the obtained results. If the expression dynamics of certain genes are compared through several cell lines, the stability of the internal control genes used should be comparable in all cell lines.

Even though we recommend careful selection and validation of reference genes for each assay and cell line, any of *YWHAZ*, *TBP*, *PPIA*, and *EF1A1* or their combination (we recommend at least two) could be used as starting reference genes in assays of osteoblastic differentiation when using other types of osteosarcoma cells or cell lines. We do not recommend the use of *ACTB* and *18S rRNA*, as they are frequently ranked as the least stable genes.

## 4. Materials and Methods

### 4.1. Cell Lines, Cell Culturing, and Cell Differentiation

The osteogenic human osteosarcoma cell lines MG-63 (ATCC^®^ CRL-1427™), HOS (ATCC^®^ CRL-1543™), and SaOS-2 (ATCC^®^ HTB-85™) were obtained from ATCC (American Tissue Culture collection; Manassas, VA, USA) and were maintained in Dulbecco’s modified Eagle’s medium (DMEM) Low Glucose (Dulbecco’s modified Eagle’s medium) (Gibco, Thermo Scientific, Waltham, MA, USA) supplemented with 10% fetal bovine serum (FBS; Gibco) and 1% L-glutamine (Gibco) at 37 °C in a humidified 5% CO_2_ atmosphere.

For osteoblast differentiation, cells were seeded in 12-well plates. Following 24 h rest, differentiation was triggered by changing to osteogenic medium: full growth medium supplemented with 100 nM Dexamethasone (Sigma, Merck, Kenilworth, NJ, USA), 50 µg/mL 2-Phospho-L-ascorbic acid trisodium salt (Sigma), and 5 mM β-Glycerophosphate disodium salt hydrate (Sigma) for MG-63 and SaOS-2 cells and full growth medium supplemented with 100 nM Dexamethasone (Sigma), 50 µg/mL 2-Phospho-L-ascorbic acid trisodium salt (Sigma), and 10 mM β-Glycerophosphate disodium salt hydrate (Sigma) for HOS cells. Medium was changed every 3–4 days. Nondifferentiated controls were maintained in full growth medium. Samples were collected at day 0 (nondifferentiated control day 0) and then every 7 days of culture to obtain five time-points (days 0, 7, 21, and 35 for HOS and MG-63; days 0, 7, 14, and 21 for SaOS-2). Nondifferentiated controls were collected on the last day of the experiment. All experiments were performed three independent times in experimental duplicates.

### 4.2. Alizarin Red S Staining

To confirm mineralization in the differentiated samples, Alizarin staining was performed. Cells were washed 3× with PBS and fixed with 4% formalin for 10 min at room temperature. Formalin was removed and the cells were washed 3× with distilled water. Cells were then stained with 2% Alizarin red S solution (pH 4.2) (Sigma) for 30 min and washed 5× with distilled water. Plates were analysed under the microscope. For spectrophotometric analysis, Alizarin was dissolved in 10% acetic acid and measured at 405 nm. Alizarin concentration was calculated from a standard curve.

### 4.3. RNA Isolation and Reverse Transcription

Total RNA was isolated using a peqGOLD Total RNA Kit (VWR, Radnor, PA, USA) following the manufacturer’s instructions. Yield and purity of RNA were determined with a NanoDrop One^C^ spectrophotometer (Thermo Scientific, Waltham, MA, USA); 3.0 µg of total RNA was reverse transcribed in a 60 µL reaction using a High-Capacity cDNA Reverse Transcription Kit (Applied Biosystems, Thermo Scientific, Waltham, MA, USA) following the manufacturer’s instructions.

### 4.4. Gene Selection, Primer Design, and Validation

The reference genes used in this study were selected based on genes frequently used for normalization in differentiation experiments on these cell lines (*ACTB*, *GAPDH*, *18S rRNA*) and genes and that were stable in studies analysing the stability of internal controls in bone-derived cells or cell lines [13,20,21,22,34,35,36,37] (Table 1). Genes and their protein products cover different functions in cells, from structural proteins to enzymes, ribosomal proteins, and different transcription factors. Primer BLAST was used for primer design. If possible, primers were selected to span an exon–exon junction, to have an optimal melting temperature around 60 °C, and to result in a PCR product length of 70–170 bp. Primers were ordered from Macrogen Europe B.V (Amsterdam, the Netherlands, EU). Primers were validated with a standard curve to determine their efficiency. The specificity of the primers was checked using melting curves.

### 4.5. RT-qPCR

RT-qPCR was performed using 5× Hot FirePol EvaGreen qPCR Supermix (Solis, BioDyne, Tartu, Estonia) following the manufacturer’s instructions. For each reaction, 2.5 ng of cDNA was used in a 15 µL reaction. The amplification programme consisted of initial denaturation at 95 °C for 15 min and 40 cycles of 95 °C for 5 s (denaturation), 60 °C for 20 s (annealing), and 20 s at 72 °C (elongation). The amplification programme was terminated with a thermal denaturing cycle to obtain the melting (thermal dissociation) curve. The RT-qPCR was performed on a LightCycler 480 (Roche Diagnostics, Rotkreuz, Switzerland). All samples were quantified in duplicates.

### 4.6. Primer Efficiency

A seven-point serial dilution of pooled cDNA samples was prepared to obtain a relative standard curve for each gene in each cell line. The standard curve was obtained by plotting the Cq (*y* axis) against the logarithm of the total cDNA concentration (*x* axis). The efficiency of each primer was calculated using Equation (1):

(1) $$\%\;E=\left(-1+10^{\left(-\frac{1}{slope}\right)}\right)\times 100$$

where *E* is primer efficiency and *slope* is a log-linear relationship between Cq values and the cDNA concentrations of the standard curve [4].

### 4.7. Data Analysis

RT-qPCR data were exported using the second derivative maximum method (LightCycler 480, software version 1.5; Roche Diagnostics, Rotkreuz, Switzerland). The stability of selected candidate genes was determined by the frequently used excel-based algorithms NormFinder (v0953) [24], geNorm (v3) [23], and BestKeeper (v1) [25].

geNorm is a Microsoft Excel-based tool which calculates gene expression stability based on the geometric mean of candidate genes’ 2^−Δ*Cq*^, calculated using Equation (2):

(2) $$2^{-\Delta Cq}=2^{min\;Cq-sample\;Cq}$$

where *min Cq* is the minimal Cq value of the sample set and *sample Cq* is the Cq of the selected sample in the sample set.

geNorm ranks candidate genes based on an internal control gene stability measure called the M value, which describes how one gene varies with respect to other candidate genes. The M value is defined as the average pairwise variation of a particular gene with all other control genes. Lower M values indicate more stably expressed genes, and genes with an M value < 1.5 are considered stable. Using stepwise exclusion of the least stable gene followed by the recalculation of the M values of the remaining genes, this algorithm can identify the two most stably expressed reference genes. In addition, geNorm calculates the pairwise variation of the selected reference genes with the others and determines how many genes are needed for accurate normalization. For this, a V value is determined between two subsequent normalization steps (V = Vn/(Vn + 1)). When V is less than 0.15, additional genes do not contribute significantly to normalization [23].

NormFinder is a model-based approach that uses 2^−Δ*Cq*^ values [Equation (2)] to estimate intra- and intergroup variations. It then combines the two to calculate a stability value, with the lowest value indicating the most stably expressed gene. Because of the model-based approach, it is less subject to gene co-regulation than the pair-wise variation approach of geNorm [24].

BestKeeper estimates inter-gene relations of candidate reference genes using pair-wise correlation analysis. It uses raw Cq and PCR efficiencies to calculate standard deviation (SD_Cq value_) and coefficient of variation (CV_Cq value_), and calculates a BestKeeper index (geometric mean) from the candidate genes’ Cqs. Then, it calculates the pair-wise correlation between each candidate gene and the BestKeeper index as the correlation coefficient. The most stably expressed genes have the lowest SD_Cq value_ and CV_Cq value_ and the highest correlation coefficient. Generally, genes are considered as stable when SD_Cq value_ < 1 [25].

To assess the overall stability of reference genes, an appropriate weight based on the ranking by each algorithm was attributed to each gene. The comprehensive ranking was performed using the geometric mean of the weights, and was calculated separately for each cell line as well as for overall stability in all cell lines combined.

Expression of differentiation markers (*ALP*, *RUNX2*, *COL1A1*) (Table 5) was evaluated by calculating the relative quantity (Rq) for each sample based on the PCR efficiency of each gene (E) using Equation (3):

(3) $$Rq=E^{\mathrm{Cq}\;\mathrm{control}-\mathrm{Cq}\;\mathrm{sample}}$$

The obtained relative quantities of marker gene expression were normalized to the relative quantity of the *ACTB* gene or to the geometric mean of the *TBP*, *PPIA*, *EF1A1*, and *YWHAZ* internal control genes. Statistical differences between normalization strategies were determined at each time point using two-way Anova (GraphPad Prism 8.0.1).

## 5. Conclusions

In this study, we identified the most stable reference genes for accurate normalization of RT-qPCR results of osteogenic differentiation of the human osteosarcoma cell lines MG-63, HOS, and SaOS-2. Using the geNorm, NormFinder, and BestKeeper programs, we identified that each cell line requires two reference genes for normalization, which are *TBP* and *PPIA* in MG-63, *YWHAZ* and *EF1A1* in HOS, and *YWHAZ* and *PPIA* in SaOS-2. Additionally, we compared our results with the outcomes of previous studies focused on identification of reference genes for other bone-derived cell lines. Our findings strongly correlate with them, confirming that *TBP*, *PPIA*, *YWHAZ*, and *EF1A1* are stably expressed during osteogenic processes in different cell types and cell lines of bone origin. Certain commonly used reference genes such as *ACTB* and *18S rRNA* are not recommended for normalization during osteogenic differentiation, as they were proven to be the least stably expressed in this study as well as in others. The internal control genes identified in this study will help researchers design better RT-qPCR experiments for the study of different processes during osteoblast differentiation of the MG-63, HOS, and SaOS-2 cell lines. Moreover, we propose a group of stably expressed genes (*TBP*, *PPIA*, *YWHAZ*, and *EF1A1*) that can be used as a starting point for further optimization of RT-qPCR experiments involving differentiation of osteoblasts in general.

## Figures and Tables

**Table 1:** Reference genes and their function and primer sequences used in this study.

Symbol Accession Number	Gene Name	Function	Sequences	Product Length	Melting Temperature (°C)
*ACTB* NM_001101.5	Actin beta	Cytoskeletal structural protein	F: 5′- CTTCGCGGGCGACGAT-3′ R: 5′- ACATAGGAATCCTTCTGACCCAT-3′	102	59.5
*GAPDH* NM_002046.7	Glyceraldehyde-3-Phosphate Dehydrogenase	Oxidoreductase in glycolysis and gluconeogenesis	F: 5′- GACAGTCAGCCGCATCTTCT-3′ R: 5′- GCGCCCAATACGACCAAATC-3′	104	60
*PPIA* NM_021130	Peptidylprolyl Isomerase A (cyclophilin A)	Cis-trans isomerization of proline imidic peptide bonds, protein folding	F: 5′- GGCAAATGCTGGACCCAACACA-3′ R: 5′- TGCTGGTCTTGCCATTCCTGGA-3′	161	64
*EEF1A1 (EF1α)* NM_001402	Eukaryotic Translation Elongation Factor 1 Alpha 1	Enzymatic delivery of aminoacyl tRNAs to the ribosome	F: 5′- CTGGACTGCATCCTACCACC-3′ R: 5′- CTCGGCCAACAGGAACAGTA-3′	106	60
*RPL13A* NM_012423.4	Ribosomal Protein L13a	Protein component of large 60S ribosomal subunit	F: 5′- GTCGTACGCTGTGAAGGCA-3′ R: 5′- GGGTTGGTGTTCATCCGCTT-3′	95	60.5
*RPLP0* NM_001002.4	Ribosomal Protein Lateral Stalk Subunit P0	Protein component of 60S ribosomal subunit	F: 5′- TCTACAACCCTGAAGTGCTTGAT-3′ R: 5′- CAATCTGCAGACAGACACTGG-3′	96	59
*TBP* NM_003194	TATA-Box Binding Protein	Component of the transcription factor IID (TFIID)	F: 5′- GCACAGGAGCCAAGAGTGAA-3′ R: 5′- TGTTGGTGGGTGAGCACAAG-3′	175	60
*18S rRNA* NR_003286	18s Ribosomal RNA	Eukaryotic small ribosomal subunit	F: 5′- GCAATTATTCCCCATGAACG-3′ R: 5′- GGCCTCACTAAACCATCCAA-3′	123	56
*HPRT1* NM_000194	Hypoxanthine phosphoribosyltransferase 1	Purine synthesis through the purine salvage pathway	F: 5′- TGACACTGGCAAAACAATGCA-3′ R: 5′- GGTCCTTTTCACCAGCAAGCT-3′	94	60
*YWHAZ* NM_145690.3	Tyrosine 3-monooxygenase/tryptophan 5-monooxygenase activation protein zeta	Cell signalling transduction through binding phosphoserine proteins	F: 5′- TGCTTGCATCCCACAGACTA-3′ R: 5′- AGGCAGACAATGACAGACCA-3′	94	59.5

**Table 2:** Primer efficiencies and correlation coefficients (R^2^) for selected internal control genes in MG-63, HOS, and SaOS-2 cell lines.

	MG-63		HOS		SaOS-2	
Gene Symbol	Efficiency (%)	R^2^	Efficiency (%)	R^2^	Efficiency (%)	R^2^
*ACTB*	101.4	0.9997	93.2	0.9991	93.0	0.9982
*GAPDH*	101.6	0.9996	94.4	0.9988	95.5	0.9999
*PPIA*	106.7	0.9975	97.1	0.9999	95.8	0.9996
*EF1A1 (EF1α)*	101.0	0.9997	99.8	0.9999	96.6	0.9998
*RPL13A*	103.5	0.9998	96.1	0.9995	96.5	0.9998
*RPLP0*	98.9	1	92.7	0.9998	92.6	1
*TBP*	103.1	0.9995	101.7	0.9999	97.4	0.9994
*18S rRNA*	99.3	0.9992	96.3	0.9999	98.8	0.9993
*HPRT1*	98.8	1	103.5	0.9956	93.4	0.9997
*YWHAZ*	100.1	0.9978	99.1	0.9968	98.9	0.9998

**Table 3:** Stability of selected candidate reference genes as ranked by geNorm, NormFinder, and BestKeeper in MG-63, HOS, and SaOS-2 cell lines. Candidates are listed from top to bottom by decreasing expression stability based on geNorm’s M value, NormFinder’s stability value, and BestKeeper’s SD, CD, and r as well as by geometrical mean of comprehensive ranking.

Cell Line	Rank	geNorm		NormFinder		BestKeeper						Comprehensive Ranking	
		Gene	M Value	Gene	Stability Value	Gene	SD (±Cq)	Gene	CV (% Cq)	Gene	Coefficient of Correlation (r)	Gene	Geomean
MG-63	1	*TBP/YWHAZ*	0.227	*PPIA*	0.079	*EF1A1*	0.259	*TBP*	1.378	*PPIA*	0.933	*TBP*	2.460
	2	*-*	-	*GAPDH*	0.086	*18S rRNA*	0.261	*RPL13A*	1.502	*HPRT1*	0.89	*PPIA*	2.631
	3	*PPIA*	0.315	*TBP*	0.094	*RPL13A*	0.282	*EF1A1*	1.576	*GAPDH*	0.831	*GAPDH*	3.288
	4	*GAPDH*	0.348	*YWHAZ*	0.118	*GAPDH*	0.326	*GAPDH*	1.825	*YWHAZ*	0.797	*YWHAZ*	3.438
	5	*EF1A1*	0.409	*EF1A1*	0.146	*TBP*	0.346	*YWHAZ*	1.834	*ACTB*	0.796	*EF1A1*	3.594
	6	*RPL13A*	0.439	*HPRT1*	0.159	*YWHAZ*	0.385	*PPIA*	2.338	*TBP*	0.775	*RPL13A*	4.816
	7	*RPLP0*	0.465	*ACTB*	0.180	*PPIA*	0.442	*18S rRNA*	2.569	*RPLP0*	0.622	*HPRT1*	6.143
	8	*18S rRNA*	0.499	*RPL13A*	0.188	*RPLP0*	0.502	*RPLP0*	2.702	*EF1A1*	0.528	*18S rRNA*	6.454
	9	*HPRT1*	0.550	*RPLP0*	0.193	*HPRT1*	0.706	*HPRT1*	2.963	*RPL13A*	0.39	*RPLP0*	7.765
	10	*ACTB*	0.645	*18S rRNA*	0.248	*ACTB*	1.011	*ACTB*	5.125	*18S rRNA*	0.063	*ACTB*	8.106
HOS	1	*HPRT1/YWHAZ*	0.216	*YWHAZ*	0.114	*EF1A1*	0.124	*EF1A1*	0.786	*HPRT1*	0.907	*YWHAZ*	2.091
	2	*-*	-	*TBP*	0.142	*18S rRNA*	0.198	*TBP*	0.992	*YWHAZ*	0.882	*EF1A1*	2.702
	3	*PPIA*	0.245	*EF1A1*	0.195	*RPLP0*	0.228	*RPLP0*	1.265	*ACTB*	0.778	*HPRT1*	2.809
	4	*TBP*	0.306	*RPLP0*	0.198	*TBP*	0.247	*YWHAZ*	1.454	*GAPDH*	0.74	*TBP*	3.288
	5	*GAPDH*	0.330	*HPRT1*	0.217	*YWHAZ*	0.309	*HPRT1*	1.764	*PPIA*	0.669	*RPLP0*	4.460
	6	*EF1A1*	0.370	*GAPDH*	0.230	*GAPDH*	0.367	*18S rRNA*	1.881	*TBP*	0.624	*GAPDH*	5.502
	7	*RPLP0*	0.400	*PPIA*	0.265	*HPRT1*	0.418	*GAPDH*	2.039	*RPLP0*	0.468	*PPIA*	5.827
	8	*18S rRNA*	0.427	*18S rRNA*	0.286	*PPIA*	0.433	*PPIA*	2.345	*EF1A1*	0.101	*18S rRNA*	5.985
	9	*ACTB*	0.478	*ACTB*	0.446	*RPL13A*	0.506	*RPL13A*	2.810	*RPL13A*	0.028	*ACTB*	7.536
	10	*RPL13A*	0.531	*RPL13A*	0.478	*ACTB*	0.666	*ACTB*	3.426	*18S rRNA*	−0.121	*RPL13A*	9.387
SaOS-2	1	*RPL13A/GAPDH*	0.212	*PPIA*	0.054	*YWHAZ*	0.209	*YWHAZ*	0.964	*PPIA*	0.920	*YWHAZ*	2.187
	2	*-*		*YWHAZ*	0.087	*TBP*	0.260	*TBP*	1.037	*RPLP0*	0.910	*PPIA*	2.402
	3	*EF1A1*	0.225	*EF1A1*	0.092	*EF1A1*	0.260	*HPRT1*	1.353	*RPL13A*	0.869	*EF1A1*	3.650
	4	*PPIA*	0.239	*RPL13A*	0.104	*PPIA*	0.323	*EF1A1*	1.531	*GAPDH*	0.858	*RPLP0*	3.882
	5	*YWHAZ*	0.260	*GAPDH*	0.114	*HPRT1*	0.325	*PPIA*	1.702	*YWHAZ*	0.832	*GAPDH*	4.183
	6	*HPRT1*	0.270	*HPRT1*	0.122	*RPL13A*	0.335	*ACTB*	1.730	*EF1A1*	0.799	*TBP*	4.789
	7	*TBP*	0.307	*RPLP0*	0.156	*ACTB*	0.359	*RPL13A*	1.879	*HPRT1*	0.739	*HPRT1*	5.194
	8	*ACTB*	0.350	*ACTB*	0.163	*GAPDH*	0.412	*GAPDH*	2.240	*ACTB*	0.718	*RPL13A*	5.785
	9	*RPLP0*	0.384	*TBP*	0.185	*18S rRNA*	0.455	*RPLP0*	2.604	*18S rRNA*	0.558	*ACTB*	7.354
	10	*18S rRNA*	0.436	*18S rRNA*	0.237	*RPLP0*	0.514	*18S rRNA*	4.080	*TBP*	0.272	*18S rRNA*	9.587

**Table 4:** Overall comprehensive ranking by geometrical mean.

Rank	Gene Name	Geomean
1	*YWHAZ*	1.587
2	*TBP*	2.884
3	*PPIA*	3.037
4	*EF1A1*	3.107
5	*GAPDH*	4.481
6	*HPRT1*	5.278
7	*RPL13A*	6.214
8	*RPLP0*	7.114
9	*18S rRNA*	8.618
10	*ACTB*	9.322

**Table 5:** Osteogenic differentiation-involved genes, their function, and the primer sequences used in this study.

Symbol Accession Number	Gene Name	Sequences	Product Length	Melting Temperature (°C)
*ALPL* NM_001127501.4	Alkaline phosphatase	F: 5′- CCAAGTACTGGCGAGACCAA-3′ R: 5′- GTGGAGACACCCATCCCATC-3′	121	60
*COL1A1* NM_000088.4	Collagen type I alpha 1 chain	F: 5′- GCCAAGACGAAGACATCCCA-3′ R: 5′- GTTTCCACACGTCTCGGTCA-3′	75	60
*RUNX2* NM_001015051.4	Runt-related transcription factor 2	F: 5′- AGCAAGGTTCAACGATCTGAGAT-3′ R: 5′- TTTGTGAAGACGGTTATGGTCAA-3′	81	59

## Data Availability

Data is contained within the article or supplementary materials.

## Supplementary Materials

The following supporting information can be downloaded at: https://www.mdpi.com/article/10.3390/ijms23084257/s1.

## References

[1] Compston J.E., McClung M.R., Leslie W.D. (2019). Osteoporosis. Lancet.

[2] Trajanoska K., Rivadeneira F. (2019). The Genetic Architecture of Osteoporosis and Fracture Risk. Bone.

[3] Zhu X., Bai W., Zheng H. (2021). Twelve Years of GWAS Discoveries for Osteoporosis and Related Traits: Advances, Challenges and Applications. Bone Res..

[4] Bustin S.A., Benes V., Garson J.A., Hellemans J., Huggett J., Kubista M., Mueller R., Nolan T., Pfaffl M.W., Shipley G.L. (2009). The MIQE Guidelines: Minimum Information for Publication of Quantitative Real-Time PCR Experiments. Clin. Chem..

[5] Drynda S., Drynda A., Feuerstein B., Kekow J., Lohmann C.H., Bertrand J. (2018). The Effects of Cobalt and Chromium Ions on Transforming Growth Factor-Beta Patterns and Mineralization in Human Osteoblast-like MG63 and SaOs-2 Cells. J. Biomed. Mater. Res. A.

[6] Go Y.Y., Kim S.E., Cho G.J., Chae S.-W., Song J.-J. (2017). Differential Effects of Amnion and Chorion Membrane Extracts on Osteoblast-like Cells Due to the Different Growth Factor Composition of the Extracts. PLoS ONE.

[7] Yu W., Zhang Y., Xu L., Sun S., Jiang X., Zhang F. (2012). Microarray-Based Bioinformatics Analysis of Osteoblasts on TiO2 Nanotube Layers. Colloids Surf. B Biointerfaces.

[8] Lan H., Hong W., Fan P., Qian D., Zhu J., Bai B. (2017). Quercetin Inhibits Cell Migration and Invasion in Human Osteosarcoma Cells. Cell. Physiol. Biochem..

[9] Chen R., Huang L.H., Gao Y.Y., Yang J.Z., Wang Y. (2019). Identification of Differentially Expressed Genes in MG63 Osteosarcoma Cells with Drug-Resistance by Microarray Analysis. Mol. Med. Rep..

[10] Fellenberg J., Dechant M.J., Ewerbeck V., Mau H. (2003). Identification of Drug-Regulated Genes in Osteosarcoma Cells. Int. J. Cancer.

[11] Czekanska E.M., Stoddart M.J., Ralphs J.R., Richards R.G., Hayes J.S. (2014). A Phenotypic Comparison of Osteoblast Cell Lines versus Human Primary Osteoblasts for Biomaterials Testing. J. Biomed. Mater. Res. A.

[12] Jeon R.H., Lee W.J., Son Y.B., Bharti D., Shivakumar S.B., Lee S.L., Rho G.J. (2019). PPIA, HPRT1, and YWHAZ Genes Are Suitable for Normalization of MRNA Expression in Long-Term Expanded Human Mesenchymal Stem Cells. Biomed. Res. Int..

[13] Studer D., Lischer S., Jochum W., Ehrbar M., Zenobi-Wong M., Maniura-Weber K. (2012). Ribosomal Protein L13a as a Reference Gene for Human Bone Marrow-Derived Mesenchymal Stromal Cells during Expansion, Adipo-, Chondro-, and Osteogenesis. Tissue Eng. Part C Methods.

[14] Hasler J., Hatt L.P., Stoddart M.J., Armiento A.R. (2020). Stable Reference Genes for QPCR Analysis in BM-MSCs Undergoing Osteogenic Differentiation within 3D Hyaluronan-Based Hydrogels. Int. J. Mol. Sci..

[15] Kraus D., Deschner J., Jäger A., Wenghoefer M., Bayer S., Jepsen S., Allam J.P., Novak N., Meyer R., Winter J. (2012). Human β-Defensins Differently Affect Proliferation, Differentiation, and Mineralization of Osteoblast-like MG63 Cells. J. Cell. Physiol..

[16] Montazeri-Najafabady N., Dabbaghmanesh M.H., Chatrabnous N., Arabnezhad M.R. (2020). The Effects of Astaxanthin on Proliferation and Differentiation of MG-63 Osteosarcoma Cells via Aryl Hydrocarbon Receptor (AhR) Pathway: A Comparison with AhR Endogenous Ligand. Nutr. Cancer.

[17] Chen Y.J., Chang M.C., Jane Yao C.C., Lai H.H., Chang J.Z.C., Jeng J.H. (2014). Mechanoregulation of Osteoblast-like MG-63 Cell Activities by Cyclic Stretching. J. Formos. Med. Assoc..

[18] Zhang Y., Andrukhov O., Berner S., Matejka M., Wieland M., Rausch-Fan X., Schedle A. (2010). Osteogenic Properties of Hydrophilic and Hydrophobic Titanium Surfaces Evaluated with Osteoblast-like Cells (MG63) in Coculture with Human Umbilical Vein Endothelial Cells (HUVEC). Dent. Mater..

[19] Rodríguez J.P., González M., Ríos S., Cambiazo V. (2004). Cytoskeletal Organization of Human Mesenchymal Stem Cells (MSC) Changes during Their Osteogenic Differentiation. J. Cell. Biochem..

[20] Stephens A.S., Stephens S.R., Morrison N.A. (2011). Internal Control Genes for Quantitative RT-PCR Expression Analysis in Mouse Osteoblasts, Osteoclasts and Macrophages. BMC Res. Notes.

[21] Abuna R.P.F., Oliveira F.S., Ramos J.I.R., Lopes H.B., Freitas G.P., Souza A.T.P., Beloti M.M., Rosa A.L. (2018). Selection of Reference Genes for Quantitative Real-Time Polymerase Chain Reaction Studies in Rat Osteoblasts. J. Cell. Physiol..

[22] Okamura K., Inagaki Y., Matsui T.K., Matsubayashi M., Komeda T., Ogawa M., Mori E., Tanaka Y. (2020). RT-QPCR Analyses on the Osteogenic Differentiation from Human IPS Cells: An Investigation of Reference Genes. Sci. Rep..

[23] Vandesompele J., de Preter K., Pattyn F., Poppe B., van Roy N., de Paepe A., Speleman F. (2002). Accurate Normalization of Real-Time Quantitative RT-PCR Data by Geometric Averaging of Multiple Internal Control Genes. Genome Biol..

[24] Andersen C.L., Jensen J.L., Ørntoft T.F. (2004). Normalization of Real-Time Quantitative Reverse Transcription-PCR Data: A Model-Based Variance Estimation Approach to Identify Genes Suited for Normalization, Applied to Bladder and Colon Cancer Data Sets. Cancer Res..

[25] Pfaffl M.W., Tichopad A., Prgomet C., Neuvians T.P. (2004). Determination of Stable Housekeeping Genes, Differentially Regulated Target Genes and Sample Integrity: BestKeeper—Excel-Based Tool Using Pair-Wise Correlations. Biotechnol. Lett..

[26] De Spiegelaere W., Dern-Wieloch J., Weigel R., Schumacher V., Schorle H., Nettersheim D., Bergmann M., Brehm R., Kliesch S., Vandekerckhove L. (2015). Reference Gene Validation for RT-QPCR, a Note on Different Available Software Packages. PLoS ONE.

[27] Schulze F., Malhan D., El Khassawna T., Heiss C., Seckinger A., Hose D., Rösen-Wolff A. (2017). A Tissue-Based Approach to Selection of Reference Genes for Quantitative Real-Time PCR in a Sheep Osteoporosis Model. BMC Genom..

[28] Ducy P., Schinke T., Karsenty G. (2000). The Osteoblast: A Sophisticated Fibroblast under Central Surveillance. Science.

[29] Langenbach F., Handschel J. (2013). Effects of Dexamethasone, Ascorbic Acid and β-Glycerophosphate on the Osteogenic Differentiation of Stem Cells in Vitro. Stem Cell Res. Ther..

[30] Ongaro A., Pellati A., Bagheri L., Rizzo P., Caliceti C., Massari L., De Mattei M. (2016). Characterization of Notch Signaling during Osteogenic Differentiation in Human Osteosarcoma Cell Line MG63. J. Cell. Physiol..

[31] Gong H., Sun L., Chen B., Han Y., Pang J., Wu W., Qi R., Zhang T.M. (2016). Evaluation of Candidate Reference Genes for RT-QPCR Studies in Three Metabolism Related Tissues of Mice after Caloric Restriction. Sci. Rep..

[32] Khan S., Roberts J., Wu S.B. (2017). Reference Gene Selection for Gene Expression Study in Shell Gland and Spleen of Laying Hens Challenged with Infectious Bronchitis Virus. Sci. Rep..

[33] Petriccione M., Mastrobuoni F., Zampella L., Scortichini M. (2015). Reference Gene Selection for Normalization of RT-QPCR Gene Expression Data from Actinidia Deliciosa Leaves Infected with Pseudomonas Syringae Pv. Actinidiae. Sci. Rep..

[34] De Lima C.A.D., de Lima S.C., Barbosa A.D., Sandrin-Garcia P., de Barros Pita W., de Azevêdo Silva J., Crovella S. (2019). Postmenopausal Osteoporosis Reference Genes for QPCR Expression Assays. Sci. Rep..

[35] Curtis K.M., Gomez L.A., Rios C., Garbayo E., Raval A.P., Perez-Pinzon M.A., Schiller P.C. (2010). EF1α and RPL13a Represent Normalization Genes Suitable for RT-QPCR Analysis of Bone Marrow Derived Mesenchymal Stem Cells. BMC Mol. Biol..

[36] Yang X., Hatfield J.T., Hinze S.J., Mu X., Anderson P.J., Powell B.C. (2012). Bone to Pick: The Importance of Evaluating Reference Genes for RT-QPCR Quantification of Gene Expression in Craniosynostosis and Bone-Related Tissues and Cells. BMC Res. Notes.

[37] Chen X., Zhang B., Zhao Y., Liu P., Zhou Y. (2013). EF1α Is a Suitable Housekeeping Gene for RT-QPCR Analysis during Osteogenic Differentiation of Mouse Bone Marrowderived Mesenchymal Stem Cells. Acta Biochim. Pol..

[38] Robledo D., Hernández-Urcera J., Cal R.M., Pardo B.G., Sánchez L., Martínez P., Viñas A. (2014). Analysis of QPCR Reference Gene Stability Determination Methods and a Practical Approach for Efficiency Calculation on a Turbot (*Scophthalmus Maximus*) Gonad Dataset. BMC Genom..

[39] Prideaux M., Wijenayaka A.R., Kumarasinghe D.D., Ormsby R.T., Evdokiou A., Findlay D.M., Atkins G.J. (2014). SaOS_2_ Osteosarcoma Cells as an in Vitro Model for Studying the Transition of Human Osteoblasts to Osteocytes. Calcif. Tissue Int..

[40] Parra-Torres A., Enríquez J., Jiménez-Ortega R., Patiño N., Castillejos-López M., Torres-Espíndola L., Ramírez-Salazar E., Velázquez-Cruz R. (2020). Expression Profiles of the Wnt/β-catenin Signaling-related Extracellular Antagonists during Proliferation and Differentiation in Human Osteoblast-like Cells. Exp. Ther. Med..

