# Supplementary material for: TBP, PPIA, YWHAZ and EF1A1 Are the Most Stably Expressed Genes during Osteogenic Differentiation
Source: Int J Mol Sci. 2022 Apr 12;23(8):4257. doi: 10.3390/ijms23084257 (PMC9025278; doi:10.3390/ijms23084257)
Supplement: Supplementary file 1 [file ijms-23-04257-s001.zip › ijms-1661292-supplementary.pdf]

Supplementary material

Selection of reference genes for RT-qPCR expression analysis in commonly used human cell lines of osteogenic origin

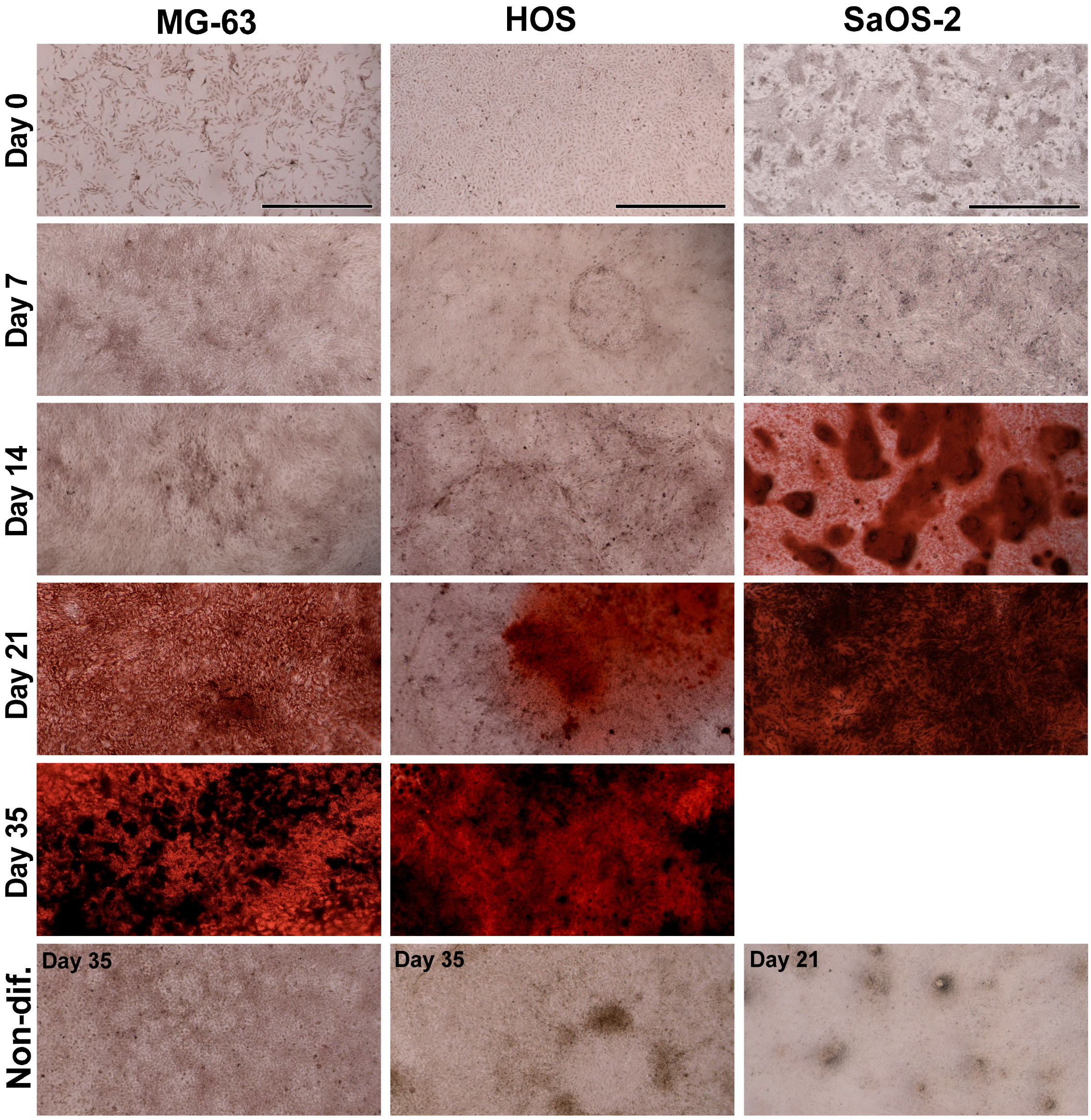

Figure S1: Photographs of Alizarin Red S stained cells at each 7th day of differentiation. Scale bars are set to 1 mm.

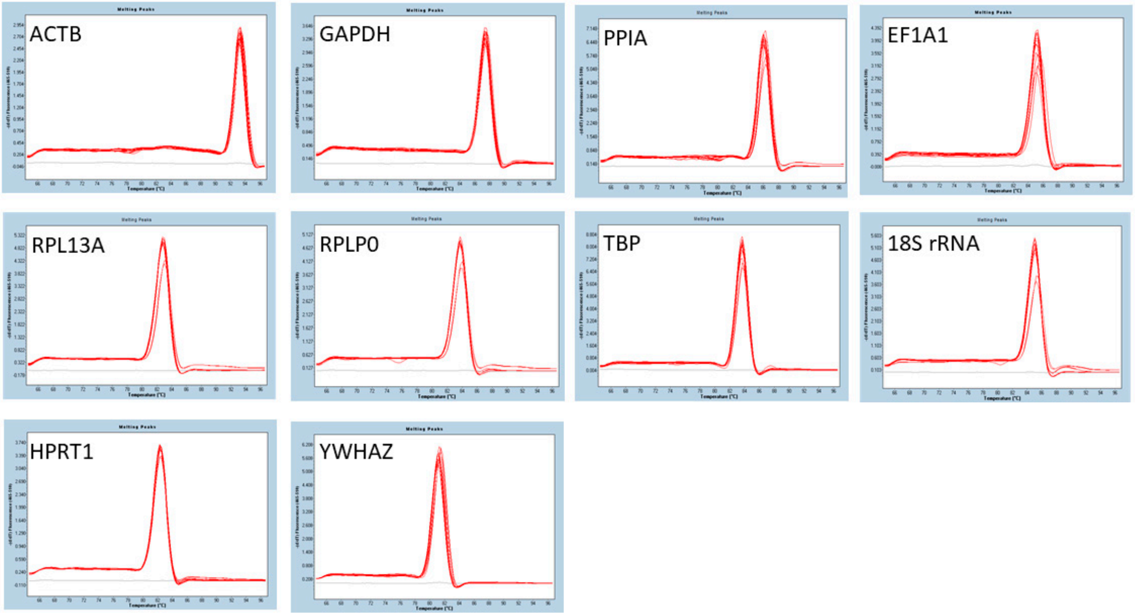

Figure S2: Primer specificity was checked by melting curves. Single curve confirms that only one product is formed. Non-treated controls are shown in grey.

Table S1: Comprehensive ranking by NormFinder.

**rank**	**overall ranking**	
-	**Gene**	**Stability value**
Best combination	GAPDH & EF1A1	0.038
1	GAPDH	0.066
2	PPIA	0.076
3	EF1A1	0.081
4	TBP	0.081
5	YWHAZ	0.101
6	ACTB	0.116
7	PRLP13A	0.121
8	HPRT1	0.123
9	PRLP0	0.153
10	18s rRNA	0.171

Table S2: Overall comprehensive ranking by geometrical mean of candidate reference genes by multiple studies .

cell type	MG-63	HOS	SaOS-2	mouse MC3T3-E1	rat MSC	ovine MSC	hiPSC	hBM-MSC (2D culture)	hBM-MSC	hBM-MSC	/	/
study reference	this study	this study	this study	(20)	(21)	(27)	(22)	(12)	(13)	(14)	/	/
name	rank	rank	rank	rank	rank	rank	rank	rank	rank	rank	geo-mean	final ranking
18S rRNA	8	8	10	5	-	-	-	7	4	7	6.733	9
ACTB	10	9	9	1	26	11	11	-	7	9	8.161	10
EF1A1	5	2	3	-	-	-	-	-	3	8	3.728	4
GAPDH	3	6	5	4	-	4	5	2	6	5	4.228	5
HPRT1	7	3	7	3	7	9	12	-	-	1	4.902	8
PPIA	2	7	2	-	5	-	-	3	-	2	3.072	1
RPL13A	6	5	8	-	-	-	7	-	1	-	4.416	6
RPLP0	9	10	4	-	-	-	-	1	-	4	4.282	7
TBP	1	4	6	-	8	-	1	4	-	6	3.337	2
YWHAZ	4	1	1	-	4	5	10	8	5	2	3.420	3
